# Post-operative management of trabeculectomy in the first three months

**Published:** 2012

**Authors:** Ian Murdoch

**Affiliations:** Senior lecturer and consultant ophthalmologist, Department of Epidemiology and International Eye Health, Institute of Ophthalmology, London, UK.

**Figure:**
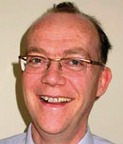
Ian Murdoch

A successful trabeculectomy is a stable surgical fistula. The one thing required to keep a fistula patent (open) is **flow**. The principal and most challenging complication is scarring; however, other complications may occur, as outlined in this article.

Prevention is vastly preferable to cure, so take every precaution you can to avoid complications. This involves careful case selection and, before surgery, optimisation of the operating environment. In addition, patients should have been counselled prior to surgery so their expectations match their post-operative experience. Nevertheless, every surgeon – even with the most meticulous attention to correct surgical approach and technique – will at some stage encounter most of the complications mentioned here.

Active interventions to avoid complications are therefore common, occurring in about half of post-operative patients at some stage.

Please note, if you have to go back to theatre with someone within four weeks of surgery, I would recommend a low threshold to general anaesthesia if at all possible. The eye is already inflamed from the first operation (and the complications). This can make the field more tricky; local anaesthetic does not work so well since it is rapidly washed away even with the use of adrenaline. In addition, the patient is all the more anxious due to the need for repeat surgery (as are you).

General anaesthesia offers a much better environment for both patient and surgeon. In addition, the operation is much faster; a ‘quick extra suture’ can be less than quick under local anaesthesia.

## Scarring

Scarring is the number one complication of trabeculectomy surgery; it takes up the majority of time in my own outpatient clinics. Management of this risk factor can improve your success rates in trabeculectomy surgery by 10% or more if you identify the patients most at risk and manage them intensively.

An appreciation of the natural history of fistula formation in trabeculectomy surgery is helpful in planning a management strategy. Immediately postoperatively, all that stands between a good result and a flat anterior chamber are your flap sutures; hence the importance of care at the time of surgery. In the weeks immediately following the procedure, scar tissue forms and offers resistance to outflow. The rate at which this tissue forms depends on ethnicity, use of antimetabolites at the time of surgery, and the external ocular environment (past medical therapy and previous trachoma, blepharitis, and conjunctival inflammation). There is often a natural peak of resistance to outflow which then resolves with remodelling. Increases in pressure are therefore not abnormal a few weeks after a trabeculectomy. The timing of this peak is very population dependent. In many African ethnic groups, it is within three to six weeks; in Caucasians it is usually about six to nine weeks post-operatively; people from Asian population groups often fall somewhere between these two. This pressure ‘hump’ may last a few weeks and (provided it does not go too high and all else looks good) observation is sufficient, since the outcome is a good, draining bleb.

**‘Scarring is the number one complication of trabeculectomy surgery’**

**Figure:**
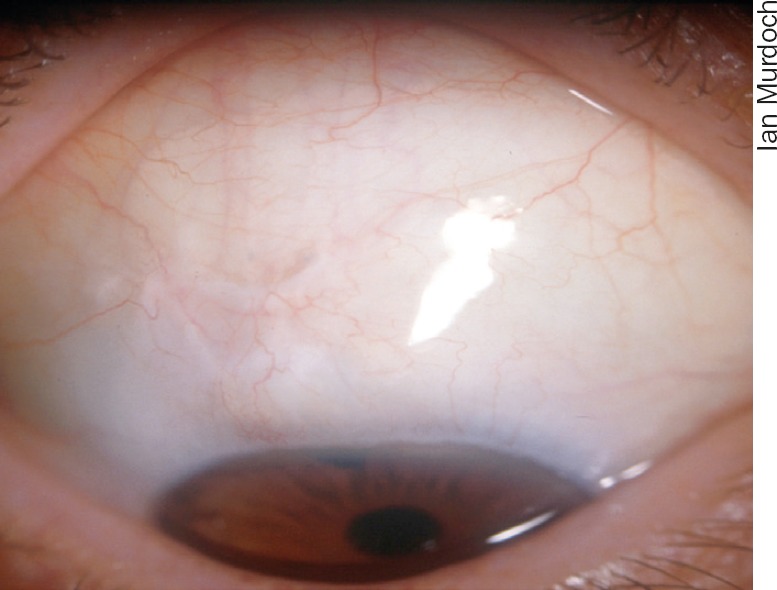
A functioning bleb

**Figure:**
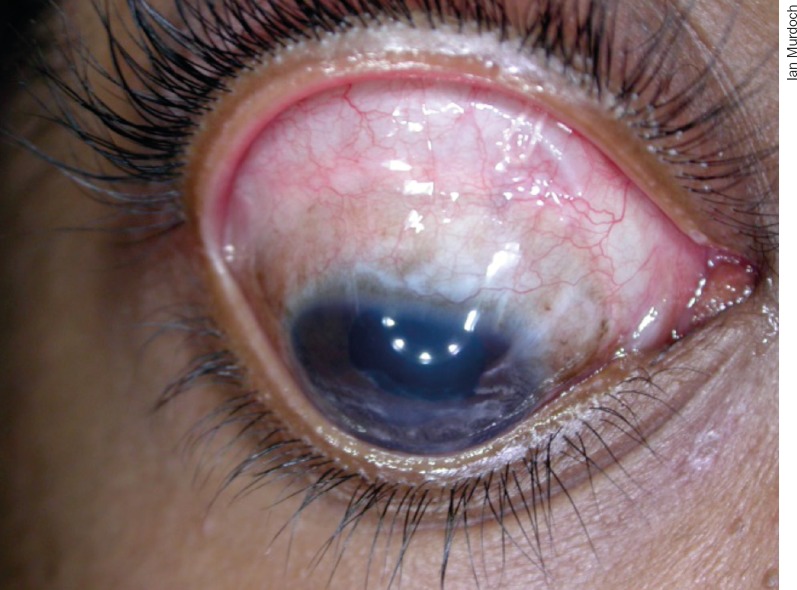
A scarred bleb – the sort to avoid if at all possible!

The best way to prevent or control scarring is frequent – a minimum of weekly – reviews. Early signs of scarring must be noticed and actively dealt with.

Mechanically re-establishing flow is the first priority. This can be achieved by massage, removing releasable sutures, dividing fixed sutures (by laser or needling) and needling of the bleb as a very last resort. Removing releasable sutures is the simplest and quickest approach by far, so give serious consideration to routine use of these sutures in your surgery.

My own personal observation is that clinicians are not usually aggressive enough in dealing with early scarring. For example, if patients have had combined trabeculectomy and cataract surgery, then the intraocular pressure (IOP) will drop naturally; however, flow needs to be established for the trabeculectomy to function. Massage techniques are outlined in the panel below, so do not give up easily!

Once you have some healing in place to offer resistance (generally a few weeks post-operatively) it is perfectly sensible to enlist the help of your patient in massaging their own eye if necessary. They can maintain flow by regular massage throughout the day whenever they put eye drops in, or even more often if you teach them carefully.

In all of this, please recognise that there is a ‘window of opportunity’ and be sensitive to the passing of this window, so you do not persist longer than appropriate. I cannot give exact timings, since some patients remain sensitive to massage over a year post-surgery, whilst others have such an intense scarring response that massage may be useless after four weeks! One final point to recognise is that poor surgical technique can result in blebs that require permanent massage. If the arms of the trabeculectomy flap do not reach to the site of the sclerostomy, then this can have the effect of creating a valve only opened with pressure posteriorly. In this instance, re-exploration and revision is the only option to achieve a satisfactory long-term result.

Steroids are the next major postoperative tool to prevent scarring. Intensive topical eye drops should be preservative free formulations if at all possible. Sub-conjunctival administration at the bleb site may, in my view, be extremely helpful. I tend to prefer administration of sub-conjunctival steroids at each post-operative visit. Finally, deposteroids, when available, should always be considered in patients who are unable or unreliable in taking their drops regularly. I personally administer these to the orbital floor rather than at the bleb site. Insufficient topical steroid therapy is, in my view, one of the principal causes of bleb scarring in patients who are subsequently referred to me for management.

**Figure 2:**
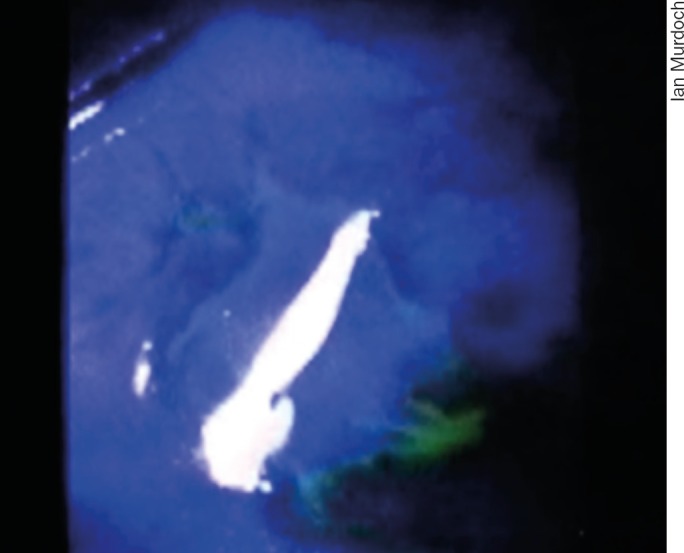
Seidel test showing leaking bleb

There is evidence that intensive use of sub-conjunctival 5FU is helpful; however, this has potential side effects. The panel on page 75 details a technique for 5FU administration. Others have reported use of other sub-conjunctival medication such as mitomycin C and anti-VEGF agents. I do not have personal experience of these.

## Conjunctival leak

Clearly, if there is a button-hole at a vital site, the conjunctiva is retracting, or the sutures are too loose. This is surgical error and needs repair. I personally like suturing the conjunctiva very precisely and carefully with at least four interrupted sutures to the limbus. There may still be a leak post-operatively during the first week or so. We have demonstrated such minor leaks to be of no consequence to final outcome.^1^ Larger leaks or persistent leaks are obviously more serious. There are two scenarios to consider, based on the IOP:

1. If the leak is combined with hypotony (low IOP) and/or a shallow anterior chamber, then this needs careful observation and surgeons should not hesitate to re-operate to correct it.
2. If the IOP is not too low and the anterior chamber is deep, then a more conservative approach can be considered for a period to see if matters resolve naturally. In this circumstance, if there is preferential anterior drainage through the leak, then sometimes releasing posterior sutures to encourage posterior drainage can solve the problem.

A long-term leak is not desirable since it is a track for infection and may be associated with instability of the anterior chamber. Repairing such a leak should be done with care and time since the track may be epithelialised. The conjunctiva should be taken down and then secured with numerous interrupted sutures. I personally make a one-third thickness groove at the limbus and use mattress sutures to secure the conjunctiva into the groove. If such repeat surgery is required, then there may well be a more vigorous scarring response, which should be adressed as above. Whilst the leak is present, the use of prophylactic antibiotics should be considered.

**Massaging techniques**

A trabeculectomy is a guarded fistula. Pushing on the guard will not achieve drainage! You need to ‘fishmouth’ the posterior opening by applying pressure to the sclera, just behind the posterior end of the scleral flap. This means the patient needs to be looking down as far as possible so you are able to apply the pressure in the correct place. Be careful not to stress the conjunctival sutures; use a slight downwards motion towards the cornea.

Self-massage by the patient should be taught using either one finger or two fingers: one from each hand. Different patients prefer different techniques. Get the patient to practice in front of you. Measure the IOP before and after the massage so you can tell patients what they have achieved.

**Figure:**
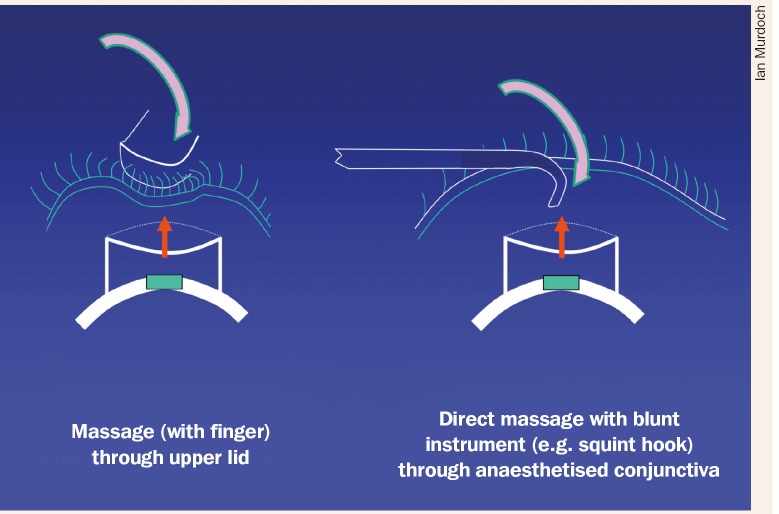

## Hypotony

Early post-operative hypotony may be seen in uveitic eyes, high myopes, and others with thin sclera where leakage through suture tracks is unavoidable. In such instances, it is usually a matter of ‘tiding over’ until the leaks seal and/or the ciliary body perks up with the postoperative steroids. ‘Tiding over’ may involve regular observation, rest (no coughing, bending, lifting or straining), and wearing a shield at night.

Should there be choroidal detachment, anterior chamber shallowing, ora threat of hypotony maculopathy, then viscoelastic or gas (isovolumetric SF6 or C3F8) may be used in the anterior chamber. This can be given at the slit lamp after anaesthesia and application of povidone iodine. If the hypotony is due to frank over-drainage, and especially if the anterior chamber is shallow/flat, then this is a surgical error and generally requires repeat surgical repair. It is better to face facts and do this early (on the first or second day after the operation) rather than late. Please remember that leaving an eye hypotonous increases the risk of suprachoroidal haemorrhage.

## Choroidal detachments

Choroidal detachments are almost always associated with hypotony, and should be evaluated from the point of prevention of hypotony. Intervention should be undertaken to prevent or resolve kissing detachments since these can be extremely destructive. Consideration of drainage or other secondary procedures are only appropriate in the extremely rare instance of exudative detachments unresponsive to medical therapy.

## Infection

Infection is, fortunately, rare. Again prevention is the key. Leaks should be observed closely, proud suture ends trimmed or removed, and any surgical intervention covered with povidone iodine (or similar) with meticulous ‘no touch’ technique. Should infection occur, admit the patient and administer intra-vitreal and intensive topical antibiotic therapy according to local protocols. Treat any local environmental predisposing factors.

## Hyphaema

This is most usual on day one, and settles quite rapidly. If it does not, then there is likely to be a bleeding diathesis, either pharmacological or pathological. Treat the bleeding diathesis first and await resolution in the eye if at all possible. In the extremely unlikely event of an eight-ball hyphaema, evacuation of the clot maybe necessary.

If available, administer tissue plasminogen activator into the anterior chamber half an hour prior to clot evacuation. This will minimise the risk of anterior segment trauma due to clot adhesion to intraocular structures.

Tranexamic acid (unless systemically contraindicated) can be given by mouth post-operatively to try to decrease re-bleeding.

## Other complications

Bleb dysaesthesia ora diffuse ‘fish eye’ bleb can persist and require bleb revision to resolve it.

Malignant glaucoma should be anticipated as a risk in short eyes (axial length <20mm, or shallow anterior chambers) and prophylactic atropine 1% prescribed pre-operatively and once daily for a minimum of three weeks post-operatively.

Malignant glaucoma is most likely in the scenario of over-drainage resulting in forward rotation of the ciliary body.

Eyes in which administration of atropine has been unsuccessful require urgent surgical intervention, which is beyond the scope of this article.

I hope the above is of some help in management strategies to improve our surgical results from this fundamental operation to prevent blindness from glaucoma. There are many other potential complications of trabeculectomy during the first three months post-operatively that space does not permit me to explore.

Please remember: prevention is better than cure, and: ‘first do no harm”!

**Sub-conjunctival 5FU administration**

Adequate anaesthetic is essential. Get the patient to look down and administer the anaesthetic drops to the upper bulbar conjunctiva (the Bells’ phenomenon ensures the rest of the eye receives anaesthetic). Allow the anaesthetic time to act. Other tips include using a cotton bud soaked in anaesthetic and lodging it under the upper lid for a period or using sub-conjunctival lignocaine and waiting an adequate time for it to disperse and work.

Enter the conjunctiva to the side and behind the scleral flap. Never inject into a bleb cyst; the forces dictate that the injection will enter the anterior chamber using the line of least resistance. (Should the 5FU enter the anterior chamber, go straight to theatre and wash the chamber out). Inject slowly; the stretch receptors produce the most discomfort, so you want to try to avoid this; and, in addition, it gives the 5FU a chance to dissipate. Once you have completed the injection do not withdraw the needle immediately but rather hold for another minute or so if possible. This gives the rest of the fluid a chance to dissipate, and prevents it leaking directly back out of your needle track onto the surface of the eye.

5FU toxicity largely comes from 5FU leaked onto the surface of the eye so it is vital to prevent this. After withdrawal of the needle, wash the eye with saline to remove any leaked 5FU.

**Figure 3:**
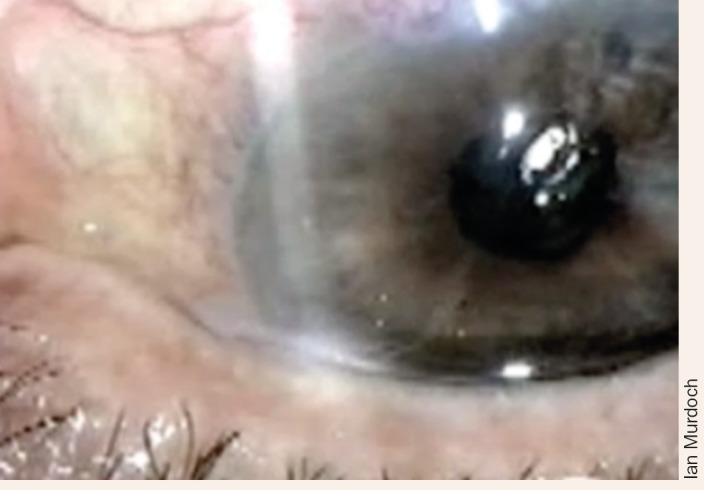
5-Flurouracil and amethocaine precipitate in the lachrymal lake, indicating that 5FU has leaked onto the ocular surface

You can check there is no 5FU by administering a drop of tetracaine (amethocaine). The pH of these anaesthetic drops is acidic (around 5) and the pH of 5FU alkaline (around 9). When the two fluids meet, they result in a white precipitate that is visible and shows the 5FU has leaked onto the ocular surface (Figure 3).
